# Varicella-zoster virus infection of the central nervous system: clinical features and proteomic analysis of cerebrospinal fluid

**DOI:** 10.3389/fcimb.2026.1835634

**Published:** 2026-06-15

**Authors:** Yu Zhang, Ruohao Li, Yanbo Zhang, Lulu Zheng, Kaixuan Bai, Xuejiao Qi, Xin Chen, Yanan Zhang, Zhijiao Song, Lantao Liang, Meiling Zhang, Hui Bu

**Affiliations:** 1 Department of Neurology, The Second Hospital of Hebei Medical University, Key Laboratory of Clinical Neurology Hebei Medical University, Shijiazhuang, China; 2 Ministry of Education, Neurological Laboratory of Hebei Province, Shijiazhuang, China; 3 Department of Neurology, Affiliated Hospital Xingtai People’s Hospital of Hebei Medical University, Xingtai, Hebei, China; 4 Department of Neurology, Hebei Chest Hospital, Shijiazhuang, China

**Keywords:** central nervous system, cerebrospinal fluid proteomics, innate immunity, neuroimmune response, neuroinflammation, varicella-zoster virus

## Abstract

**Objective:**

Varicella-zoster virus (VZV) involvement of the central nervous system (CNS) can cause severe complications; however, the underlying pathogenic mechanisms remain incompletely understood. This study describes the clinical characteristics of VZV CNS infection and explores the molecular mechanisms involved through cerebrospinal fluid (CSF) proteomic analysis.

**Methods:**

This study included 69 patients diagnosed with VZV CNS infection at our center. Their clinical symptoms, laboratory tests, and neuroimaging results were analyzed. CSF samples from nine patients with VZV CNS infection (VZV group) and 10 controls without CNS infection (Ctrl group) were subjected to proteomic analysis.

**Results:**

The most common clinical manifestations were headache (79.7%), fever (56.5%), and motor/sensory disturbances (30.4%). Neuroimaging revealed abnormal brain parenchyma in 18.8% of the cases. CSF from most patients showed elevated white blood cell counts (0–1400 × 10^6^/L) and protein levels (0.11–7.61 g/L), and elevated cerebrospinal fluid pressure (60–330 mmH_2_O). Proteomic analysis indicated the number and abundance of CSF proteins to be markedly higher in the VZV group than in the Ctrl group. Up-regulated proteins in the VZV group were primarily associated with type I interferon signaling, pyroptosis, and increased blood–brain barrier permeability. Gene Ontology enrichment analysis indicated that upregulated proteins were predominantly associated with innate antiviral immunity. Wikipathways were enriched for lymphocyte activation and inflammatory signaling pathways.

**Conclusion:**

This study integrates clinical and proteomic analyses to reveal the clinical and molecular features of VZV CNS infection. Synergistic over-activation of the type I interferon response, inflammatory signaling, and lymphocyte activation drives a robust neuroinflammatory reaction, which may underlie blood–brain barrier disruption and neurological deficits. Furthermore, over-activation of the complement system in severe encephalitis provides new insights for understanding disease severity and potential therapeutic targets. These findings underscore the potential of this approach for developing diagnostic markers and targeted therapeutic strategies.

## 1 Introduction

Varicella-zoster virus (VZV) is a neurotropic DNA virus classified within the α-herpesvirus family. It primarily enters the human body through the respiratory tract, then spreads to T lymphocytes in the bloodstream via the lymphoid tissue in the pharynx (Gershon et al., 2010). Transmission can also occur through direct contact, and the incubation period is generally 10–21 days. The primary infection usually manifests on the skin with characteristic vesicular rashes (Andrei and Snoeck, 2021). Following the initial infection, VZV can establish latency infection in the dorsal root, cranial root, and autonomic ganglia (Boucher et al., 2017). When VZV-specific immunity declines, triggers, such as advanced age, immunosuppressive conditions, or physiological or psychological stress, may lead to reactivation and replication of the latent virus. This most often results in herpes zoster, also known as shingles, and associated neuralgia along the path of a unilateral (or, rarely, bilateral) peripheral sensory nerve (Yan et al., 2022; Grahn and Studahl, 2015). Both immunocompromised individuals and those with normal immunity are susceptible to peripheral nerve damage, such as post-herpetic neuralgia, cranial nerve involvement, Guillain-Barré syndrome, and even central nervous system (CNS) dysfunction, including meningitis, meningoencephalitis, encephalitis, myelopathy, and vasculitis (Omland et al., 2021; DeBiasi and Tyler, 2004). Among these CNS complications, meningitis and meningoencephalitis occur most commonly (Herlin et al., 2021).

The global incidence of herpes zoster is reported to be 3–5 cases per 1,000 person-years in the general population (Tyrberg et al., 2025), while the incidence in the Asia-Pacific region is higher, ranging from 3 to 10 cases per 1,000 person-years (Chen et al., 2017), with an annual increase of 2.5% to 5.0% (Kawai et al., 2016). The mortality rate is between 0.017 and 0.465 per 100,000 person-years, and the recurrence rate ranges from 1% to 10% (Chen et al., 2017; Kawai et al., 2016; Tseng et al., 2020). The incidence of herpes zoster is age-dependent, with the incidence in elderly individuals (aged ≥ 65 years) ranging from 3.9 to 11.8 per 1,000 person-years (Patil et al., 2022; van Oorschot et al., 2021). In addition to age, VZV CNS infection is also associated with immune impairments resulting from organ transplantation, autoimmune diseases, cancer, or immunosuppressive therapies (Gauthier et al., 2009; Gilden et al., 2000).

Historically, VZV infection was considered to have mild symptoms with a favorable prognosis. However, recent studies have suggested it may be associated with poorer outcomes (Yan et al., 2022). A 2021 epidemiological study based on large-scale U.S. health insurance data confirmed that the risk of developing Guillain-Barré Syndrome significantly increases within 1 to 42 days following the onset of herpes zoster, with a hazard ratio of 6.3 for individuals aged 18–64 years and 4.1 for those aged 65 years or older (Anderson et al., 2021). A study published in 2025 by Fullerton et al. showed that approximately 10% of cases of arterial ischemic stroke were associated with recent asymptomatic VZV reactivation, suggesting that the virus may contribute to stroke pathogenesis, particularly during periods of weakened immunity (Fullerton et al., 2025). Josephson et al. indicated that primary varicella-zoster virus infection can lead to venous thrombus formation. This may be related to direct endothelial cell damage, vascular inflammation, the production of anti-protein S autoantibodies, or pre-existing hypercoagulable states. This phenomenon is referred to as varicella-associated autoantibody syndrome (Josephson et al., 2001).

The clinical symptoms, imaging manifestations, and cerebrospinal fluid (CSF) findings of VZV-associated encephalitis or meningitis are often nonspecific and can closely resemble those caused by other pathogens, such as herpes simplex virus or *Myobacterium tuberculosis*. Traditional diagnostic methods, such as viral culture, are time-consuming and have low sensitivity. Meanwhile, antibody testing can be prone to delayed responses and cross-reactivity, and can lead to false-negative and false-positive diagnoses. The development of molecular biology techniques, such as PCR (polymerase chain reaction) and mNGS (metagenomic next-generation sequencing), has significantly improved the detection rate of VZV. However, PCR may yield false-negative results in late-stage cases or when the viral load is low because of DNA degradation (Liu et al., 2025). mNGS is particularly useful in cases of low viral load or mixed infection, and it significantly outperforms traditional methods in detecting pathogens in complex or atypical cases. Furthermore, mNGS allows for the simultaneous exclusion of other potential interfering pathogens. Since the introduction of mNGS as a method for pathogen detection in CSF, the detection rate of VZV has notably increased, leading to a deeper understanding of VZV infection.

Although the diagnostic rate has been greatly improved by mNGS, significant sequelae remain after antiviral treatment. However, the marked cell-associated nature of VZV, coupled with its strict species-specificity, has constrained research on its pathogenesis. As a result, studies on the immunobiology of VZV infection have primarily focused on human clinical samples. The host’s innate and adaptive immune responses are crucial in controlling disease severity and inhibiting VZV reactivation (Laing et al., 2018). Subsequent to recognition by the innate immune system, the virus can activate key transcription factors, leading to the induction of type I interferons (IFNα/β) and pro-inflammatory cytokines (Paludan et al., 2011). The adaptive immune response is more complex, with T cells and B cells playing pivotal roles in managing primary VZV infection, establishing latency, and preventing reactivation (Laing et al., 2018). Notably, Activated VZV-specific CD4+ T cells display expression of the skin-homing receptor cutaneous lymphocyte-associated antigen (CLA) during the early phase of varicella, which aligns with the role of effector T cells migrating to the skin to clear VZV (Malavige et al., 2008). Proteomic studies of patients with VZV meningitis show significant up-regulation of inflammatory proteins (e.g., CXCL10, IL1RN) and down-regulation of neuro−metabolic proteins (e.g., CKMT1B, SLITRK3). Elevated levels of CSF interleukin (IL)-18 in poor−outcome cases indicate its potential role as a prognostic biomarker and highlight inflammation as a key mechanism in VZV CNS infection (Liu et al., 2023).

In our study, we applied proteomics to further explore protein biomarkers associated with VZV CNS infection. By analyzing the biological and immunological characteristics of such biomarker proteins, key pathogenic pathways and targets with potential diagnostic or prognostic value can be investigated. We predict that this approach will enhance our understanding of VZV-related neurological diseases and improve diagnostic capabilities.

## 2 Materials and methods

### 2.1 Study population and diagnostic criteria

Sixty-nine patients with VZV encephalitis/meningitis who were treated in our hospital from June 2018 to July 2025 were enrolled in this study. All patients had a confirmed microbiological diagnosis of VZV CNS infection via CSF mNGS. VZV positivity by mNGS was defined as the detection of at least three reads corresponding to VZV-specific sequences (Miller et al., 2019), mapped to distinct regions of the viral genome, exceeding background levels, and interpreted in the context of compatible clinical features (Gu et al., 2019; Wilson et al., 2019). Their clinical symptoms, laboratory tests, and neuroimaging results were analyzed.

### 2.2 CSF collection and storage

CSF samples were collected at initial clinical evaluation after symptom onset and prior to the initiation of targeted therapy. Lumbar punctures were performed as part of the diagnostic workup, and samples were obtained concurrently for metagenomic mNGS.

All CSF samples were processed following standardized pre-analytical protocols, including immediate centrifugation to remove cells and debris, aliquoting to avoid repeated freeze–thaw cycles, and storage at −80 °C until analysis. These procedures were implemented to minimize pre-analytical variability, as delayed processing, temperature fluctuations, and repeated freeze–thaw cycles have been shown to significantly affect the CSF proteome and mass spectrometry results (Teunissen et al., 2009; Rosenling et al., 2009).

### 2.3 Peptide preparation and quantification

Proteins were enriched from 40 μL CSF samples using a magnetic bead–based enrichment approach according to the manufacturer’s instructions. Briefly, samples were incubated with binding buffer and enrichment reagents, followed by magnetic separation and repeated washing to obtain purified protein-bound beads.

The enriched proteins were subjected to on-bead enzymatic digestion using a two-step protocol. The resulting peptides were collected after magnetic separation, desalted using solid-phase extraction columns, and dried under vacuum. Peptides were subsequently reconstituted in MS loading buffer, and peptide concentrations were determined using a NanoDrop spectrophotometer (Thermo Scientific) prior to mass spectrometry analysis.

### 2.4 Data-independent acquisition mass spectrometry

Peptides were spiked with iRT standards (1:20, v/v). Separation was performed on a Vanquish Neo UHPLC system (Thermo Scientific) equipped with a PepMap Neo C18 column (75 μm × 250 mm, 2 μm) at 50 °C and 300 nL/min. Mobile phases were 0.1% formic acid in water (A) and acetonitrile (B). Linear gradient: 4–99% B in 6.9 min.

Eluted peptides were analyzed on an Orbitrap Astral mass spectrometer (Thermo Fisher) in DIA mode. MS¹: resolution 240,000 at m/z 200, scan range 380–980 m/z (AGC 500%, max IT 5 ms). HCD fragmentation (NCE 25%). MS²: scan range 150–2000 m/z using 60 variable windows (2 m/z) over 380–980 m/z (AGC 500%, max IT 3 ms). Cycle time: 0.6s.

### 2.5 Protein identification, quantification, and bioinformatic analysis

Raw DIA data were processed using Spectronaut Pulsar (version 18.7, Biognosys) for protein identification and label-free quantification, with the UniProt human reference proteome (uniprot-Homo sapiens-9606-2024.2.1.fasta) used as the search database. Database searching was performed against the human reference proteome, and protein identification required at least one unique peptide.

Proteins were further filtered to retain those with sufficient data completeness (valid values in at least 50% of samples within at least one group). Missing values were imputed using a combination of group-wise mean imputation and minimal value replacement, followed by median normalization and log2 transformation.

Principal component analysis (PCA) was performed to evaluate data quality and sample clustering. Differentially expressed proteins were identified based on a fold-change threshold (≥ 2.0 or ≤ 0.5) and *P* < 0.05. All identified proteins were annotated using Gene Ontology (GO; http://www.blast2go.com/b2ghome; http://geneontology.org/) and WikiPathways (https://www.wikipathways.org/).

In addition, hierarchical clustering was used to visualize the expression patterns of immune-related proteins. Weighted gene co-expression network analysis (WGCNA) was performed to identify modules of co-expressed proteins. Proteins derived from selected modules were further explored, and their expression patterns across clinical subgroups were visualized using hierarchical clustering and heatmaps in an exploratory manner.

## 3 Results

### 3.1 Clinical characteristics of enrolled patients

Among the 69 enrolled patients, 31 were men and 38 were women. The mean age was 53.06 ± 14.60 years (range: 19–83 years). Most patients (71.01%) no pre-existing physical or cognitive impairments at the time of admission. Nineteen patients (27.54%) were immunocompromised due to immunosuppressive therapies such as prednisone or other immunosuppressive drugs (4), diabetes mellitus (12), or solid malignancies (4). None of the patients were found to have a primary immunodeficiency. Patients predominantly presented with headache (79.71%), fever (56.52%), extremity motor/sensory deficits (30.43%), or cranial nerve palsy (26.09%). Other frequent clinical manifestations included confusion (17.39%), personality changes (14.49%), seizures (preceding or at admission) (11.59%), cerebellar ataxia (5.80%), and dysuria (5.80%). In addition, 13 patients experienced herpes zoster on the skin of the head and face (26.64%), while 10 patients had herpes zoster on the trunk (17.39%). Lumbar puncture was performed on all enrolled patients to measure CSF opening pressure. The pressure was within the normal range (80–180 mmH2O) in 40 patients, mildly elevated (180–330 mmH2O) in 20 patients, and markedly elevated (> 330 mmH2O) in 5 patients. Data on pressure were not available for 4 patients. Routine CSF examination revealed elevated leukocyte counts in the majority of cases that were classified as mild (8–100 × 106/L) in 18 patients, moderate (100–500 × 106/L) in 36 patients, and severe (> 500 × 106/L) in 9 patients. Differential counts showed a predominance of mononuclear cells, accounting for approximately 97%–100% of total cells. Biochemical analysis indicated elevated CSF protein levels, which were within the normal range (0–0.45 g/L) in 18 patients, moderately elevated (0.45–1 g/L) in 23 patients, and severely elevated (> 1 g/L) in 27 patients. In the majority of patients, glucose and chloride concentrations in the CSF were within normal ranges (**Table 1**).

**Table 1:** Demographic, clinical, and paraclinical characteristics of 69 patients with varicella-zoster virus (VZV) central nervous system infection.

Variable	Obs	N (%)/median (IQR)
Age (y)	69	53.06 (39-63)
Male gender		31 (44.93%)
Comorbidity/immunosuppression		
Diabetes mellitus		12 (17.39%)
Prednisolone (any dosage)		4 (5.80%)
Solid cancer		4 (5.80%)
Clinical presentation		
Headache		55 (79.71%)
Fever (≥38.0°C)		39 (56.52%)
Confusion		12 (17.39%)
Personality changes		10 (14.49%)
Seizures (preceding or at admission)		8 (11.59%)
Cranial nerve palsy		18 (26.09%)
Extremity motor/sensory deficits		21 (30.43%)
Cerebellar ataxia		4 (5.80%)
Dysuria		4 (5.80%)
Site of herpes zoster		
Head and face		17 (24.64%)
Trunk		12 (17.39%)
Cerebrospinal fluid analysis		
High Pressure (≥180mmH2O)		29 (42.03%)
WBC count (×10^6^/L)		244.94 (30-330)
Protein (g/L)		1.18 (0.44-1.27)
Abnormal imaging		
Brain		13 (18.84%)
Spinal cord		6 (8.70%)
Cranial nerve		4 (5.80%)
Haemorrhage		1 (1.45%)
Contrast enhancement		7 (10.14%)
Modified rankin Scale (at admission)		
2-3		52 (75.36%)
4-5		17 (24.63%)
Modified rankin scale (at discharge)		
0-3		63 (91.30%)
4-5		6 (8.70%)

**Figure 1:**
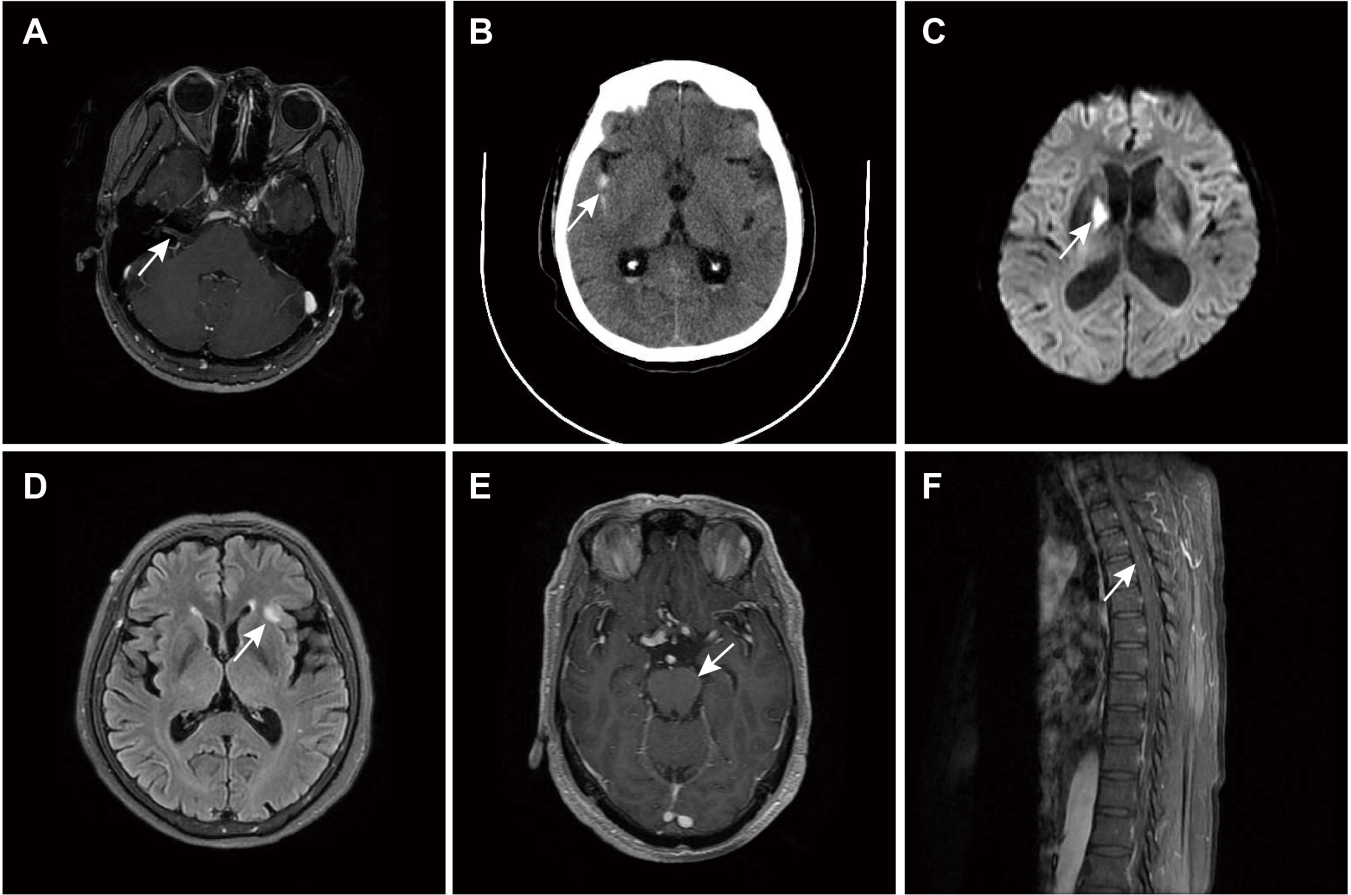
Imaging of a patient with VZV central nervous system infection. **(A)** Enhancement of the facial and vestibulocochlear nerves **(B)** Subarachnoid hemorrhage **(C)** Subacute infarction of the right basal ganglia **(D)** Inflammatory lesion of the left insular cortex **(E)** Superficial leptomeningeal enhancement of the brainstem **(F)** Myelitis at the T8–T11 vertebral levels.

Among the patients who underwent cranial magnetic resonance imaging (MRI), 13 presented with brain abnormalities (18.84%), 6 had spinal cord injuries (8.70%), 4 exhibited cranial nerve damage (5.80%), and 1 had subarachnoid hemorrhage (1.45%), with 7 of these patients showing contrast enhancement (10.14%) (**Table 1**; **Figure 1**).

Modified Rankin Scale (mRS) scores were assessed both at admission and discharge. At admission, 52 patients (75.36%) presented with moderate disability (mRS 2–3), while 17 patients (24.63%) exhibited severe disability (mRS 4–5). At discharge, clinical outcomes improved significantly, with 63 patients (91.30%) achieving a favorable functional status (mRS 0–3), and only six patients (8.70%) remaining with severe disability (mRS 4–5) (**Table 1**).

### 3.2 Proteomic profiling and identification of differentially expressed proteins

**Figure 2:**
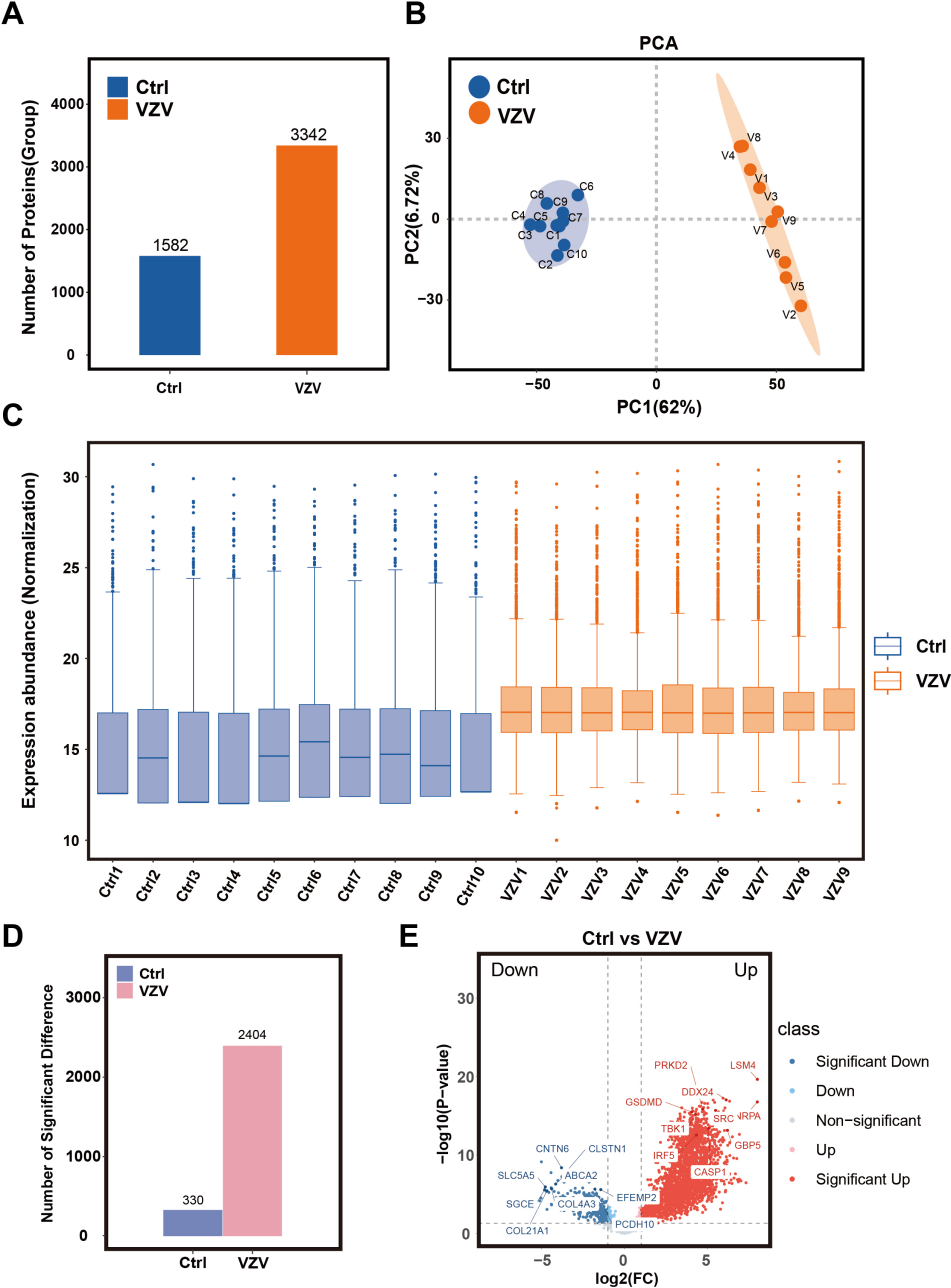
Summary of the analysis and comparison of protein expression in different groups. **(A)** Proteins with ≥50% expression frequency were considered identified in the respective group, with identification results shown for different groups. **(B)** Principal component analysis (PCA) of the expression levels of identified proteins, illustrating the relationships between samples from different dimensions. **(C)** Boxplot and density plot of the expression levels of reliable proteins. **(D)** Differential protein analysis between two groups, with differential selection criteria: P-value < 0.05. **(E)** Evaluation of protein expression differences between groups, with differential selection criteria: P-value < 0.05, FC ≥ 2.0 or FC ≤ 1/2.0.

A total of 3,342 proteins were identified in the VZV group and 1,582 in the Ctrl group. The protein content and number of protein species were markedly higher in the VZV group than in the Ctrl group (**Figures 2A, C**). Proteins exhibiting differential expression between VZV group samples and Ctrl group samples were significant (**Figure 2B**). We analyzed the differentially expressed proteins between the two groups, and identified 2,404 proteins as up-regulated and 330 proteins as down-regulated in the VZV group compared with the Ctrl group (**Figure 2D**). The up-regulated proteins were mainly associated with the type I interferon pathway (McNab et al., 2015) (TBK1, IRF5, and GBP5), pyroptosis (Shi et al., 2017) (CASP1 and GSDMD) and proteins related to increased blood–brain barrier permeability (Weiss et al., 2009) (SRC and PRKD2). The down-regulated proteins were mainly associated with neuronal synaptic connectivity and adhesion (Südhof, 2018) (CNTN6, CLSTN1, and PCDH10), extracellular matrix structural homeostasis (Theocharis et al., 2016) (COL4A3, COL21A1, and EFEMP2) and myelin homeostasis and ion homeostasis (Nave and Werner, 2014) (ABCA2, SGCE, and SLC5A5) (**Figure 2E**).

### 3.3 Functional enrichment analysis of differentially expressed proteins

**Figure 3:**
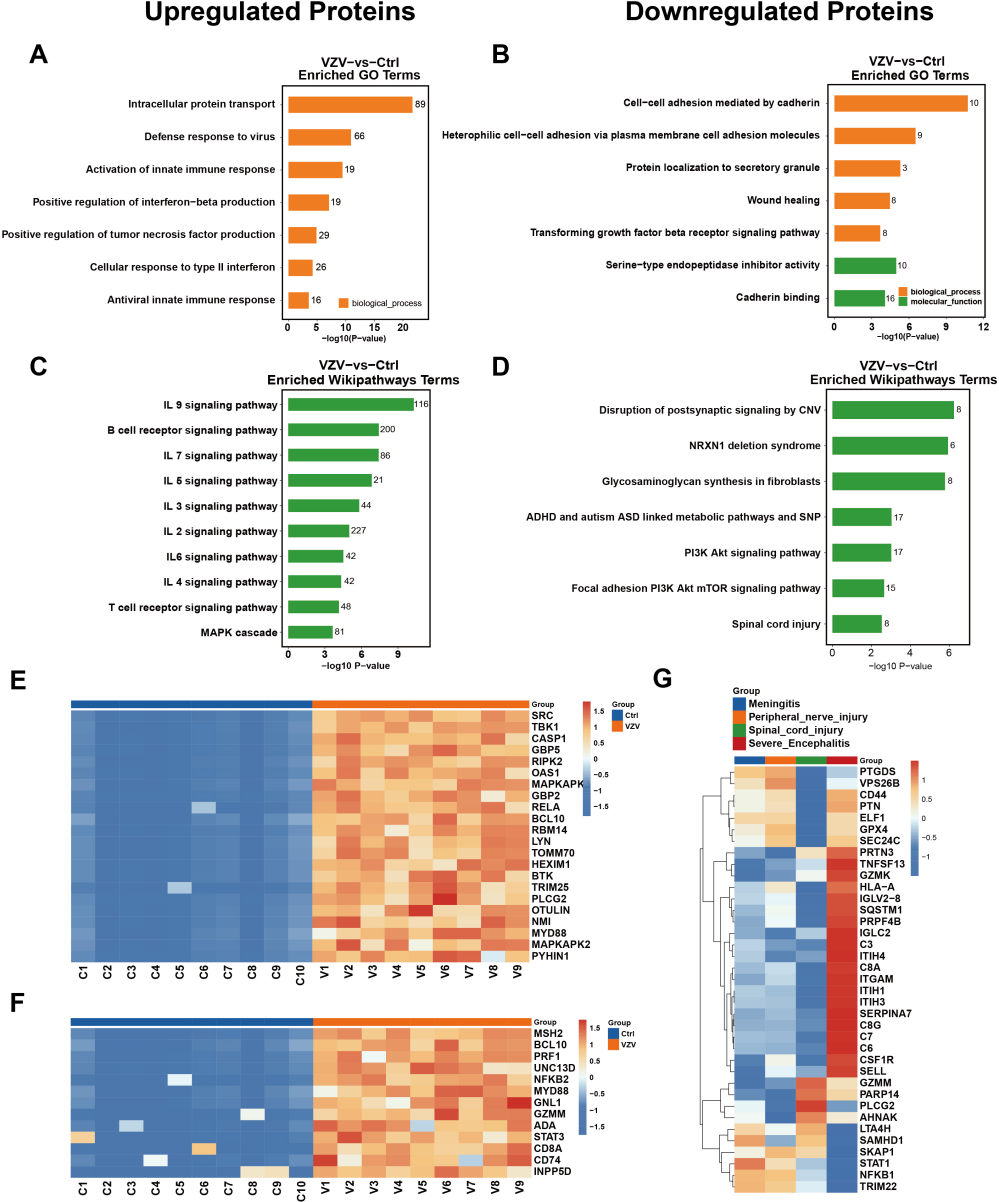
**(A)** GO enrichment analysis of upregulated proteins in the VZV group. **(B)** GO enrichment analysis of downregulated proteins in the VZV group. **(C)** Wikipathway enrichment analysis of upregulated proteins in the VZV group. **(D)** Wikipathway enrichment analysis of downregulated proteins in the VZV group. **(E)** Proteins related to innate immunity. **(F)** Proteins related to adaptive immunity **(G)** Comparison between groups across the four sample sets.

We performed enrichment analysis of the differentially expressed proteins using GO and Wikipathways. GO enrichment analysis revealed that the up-regulated pathway proteins were primarily involved in the innate antiviral immune response, with enhanced levels in the categories of intracellular protein transport, defense response to virus, antiviral innate immune response, positive regulation of interferon beta production, positive regulation of tumor necrosis factor production, cellular response to type II interferon, and activation of innate immune response. This pattern strongly indicates a sustained antiviral inflammatory state within the CNS. Down-regulated pathways were significantly enriched for diminished intercellular adhesion, including cell-cell adhesion mediated by cadherin and cadherin binding, as well as heterophilic cell-cell adhesion, protein localization to secretory granule, wound healing, transforming growth factor beta receptor signaling, and serine-type endopeptidase inhibitor activity (**Figures 3A, B**). Significantly up-regulated Wikipathways were associated with lymphocyte activation and inflammatory signaling pathways, including T/B cell receptor signaling pathways, and comprehensive up-regulation of cytokine signaling pathways (e.g., IL-2/3/4/5/6/7/9). Down-regulated pathways, were enriched in postsynaptic signaling, disruption of postsynaptic signaling, NRXN1 deletion syndrome, glycosaminoglycan synthesis, and spinal cord injury (**Figures 3C, D**).

Compared with the Ctrl group, levels of innate immune-related proteins (SRC, TBK1, CASP1, GBP5, RIPK2, OAS1, MAPKAPK3, GBP2, RELA, BCL10, RBM14, LYN, TOMM70, HEXIM1, BTK, TRIM25, PLCG2, OTULIN, NMI, MYD88, MAPKAPK2, and PYHIN) and adaptive immune-related proteins (MSH2, BCL10, PRF1, UNC13D, NFKB2, MYD88, GNL1, GZMM, ADA, STAT3, CD8A, CD74, and INPP5D) were elevated in the VZV group (**Figures 3E, F**).

### 3.4 Subgroup analysis based on clinical manifestations

All VZV group patients were grouped according to clinical symptoms and could be divided into a Meningitis group (4 cases), a Peripheral nerve injury group (3 cases), a Spinal cord injury group (1 case) and a Severe encephalitis group (1 case) (**Figure 3G**). We compared specimens among the four groups and found the up-regulated proteins in the Meningitis group to be STAT3, SAMHD1, NFKB2, and TRIM22, indicating a mechanism related to the inhibition of viral replication and inflammatory signaling. The up-regulated proteins in the Peripheral nerve injury group were VPS26B, PTGDS, and SKAP1, which are associated with anti-inflammation and promotion of nerve myelin repair and regeneration. The proteins up-regulated in the Spinal cord injury group were PLCG2, GZMM, PARP14, and AHNAK, which are associated with regulation of immune cell function, clearing of infected cells, maintenance of cell morphology, and injury repair. The proteins up-regulated in the Severe encephalitis group were mainly associated with complement-related proteins, including C3, C8A, C8G, C7, C6, and ITGAM, but also included IGLC2, ITIH4, ITIH1, ITIH3, SERPINA7, TNFSF13, and GZMK, indicating over-activation of the complement system. This can result in irreversible cell lysis and death, and can activate B-cells by exacerbating the inflammatory response. Proteins such as ITIH1, ITIH3, and ITIH4 were also up-regulated indicating severe blood–brain barrier disruption (**Figure 3G**).

## 4 Discussion

### 4.1 Clinical characteristics of VZV CNS infection

In this study, we conducted a retrospective analysis of clinical data from 69 patients with VZV CNS infection and combined them with CSF proteomic analysis to reveal the clinical characteristics of VZV CNS infection and its underlying molecular mechanisms. The cohort of patients with VZV meningitis described in this study was predominantly middle-aged to elderly (mean 53.1 years) with no significant sex bias, consistent with the epidemiological profile of VZV CNS infection summarized by Nagel et al (Nagel et al., 2020). Although 28.99% of patients had underlying immunosuppressive conditions, the vast majority (71.01%) were immunocompetent, aligning with the observation by Grahn & Studahl (Grahn and Studahl, 2015) that severe CNS infection resulting from VZV reactivation is not uncommon in immunocompetent hosts. The clinical presentation of the patients with VZV meningitis was dominated by headache and fever, with CSF showing typical lymphocytic-predominant pleocytosis and elevated protein levels, while glucose and chloride levels were mostly normal. This pattern closely matches the characteristics of VZV meningitis reported by Hong et al (Hong et al., 2014), further supporting this profile as typical for viral meningitis. Notably, the rate of abnormal cranial MRI findings in our cohort (18.84%) was lower than that reported by Persson et al (Persson et al., 2009). (by approximately 30%), which may reflect differences in imaging indications or disease severity across populations. Importantly, functional outcomes were significantly improved at discharge, with the vast majority achieving good recovery, underscoring the critical role of early clinical recognition and intervention. Our findings validate the key clinical and laboratory features reported in key prior studies and highlight the importance of vigilance for neurological complications of VZV reactivation even in the general population in the absence of severe immunodeficiency.

### 4.2 Study design and selection of proteomic cohort

A subset of samples from the aforementioned cohort, including CSF from nine patients with VZV CNS infection and ten patients with non-infectious neurological disorders, was selected for proteomic analysis. A marked difference in CSF protein profiles was observed between the two groups, with both the overall protein abundance and the number of detectable proteins being higher in the VZV group.

The control group consisted of patients with non-infectious neurological conditions, whose CSF biochemical parameters, opening pressure, and routine analyses were consistent with non-infectious etiologies. Although these controls may differ from healthy individuals, they represent a clinically relevant comparison group distinct from infectious CNS diseases. Differential expression analysis between the two groups may therefore provide preliminary insights into the proteomic characteristics associated with VZV CNS infection.

Compared with previous studies of CSF biomarkers in viral CNS infections, several aspects of our study may add to the current understanding. First, most prior investigations have focused on a limited number of predefined biomarkers, such as cytokines (e.g., IL-6, CXCL10) or neuronal injury markers (e.g., NfL, tau), or on specific immune cell features, while only a few studies have explored omics-level changes, often with limited proteome depth or heterogeneous viral cohorts (Liu et al., 2023; Kuhn et al., 2018; Schub et al., 2018; Tyrberg et al., 2020; Ahmed et al., 2022; Grahn et al., 2013). In contrast, we applied an unbiased DIA-based proteomic approach to systematically characterize the CSF protein landscape. Second, proteomic studies specifically addressing VZV CNS infection remain limited, and comprehensive characterization of CSF protein alterations in this setting has not been well established^4^. Third, by integrating proteomic analysis with a relatively large clinical cohort, our study provides a combined clinical–molecular perspective on VZV CNS infection.

### 4.3 Global proteomic alterations and biological implications

Our proteomic analysis systematically revealed protein changes in CSF related to immune activation, cell death, blood–brain barrier disruption, and neurostructural damage caused by VZV infection. Proteomic analysis showed that the types and amounts of proteins in the CSF of the VZV-infected group were markedly elevated compared with those of the control group, indicating that extensive changes in protein abundance may be closely related to the immune and inflammatory responses of the CNS. The protein content in the VZV group was elevated compared with that of the Ctrl group, and the types of proteins were different. In the VZV group, the type I interferon pathway (e.g., TBK1 and IRF5), cellular pyroptosis death-associated proteins (e.g., CASP1 and GSDMD), and blood–brain barrier permeability-related proteins (e.g., SRC and PRKD2) were significantly up-regulated. This is consistent with previously reported mechanisms of innate immune response activation by VZV (Paludan et al., 2011; Wang et al., 2005). However, given the limited sample size and observational design, these results should be considered exploratory and require validation in larger cohorts.

### 4.4 Immune activation and pathway enrichment analysis

GO enrichment analysis indicated that the up-regulated pathway proteins were primarily focused on innate antiviral immune responses, and that the innate immunity-related proteins were elevated in the VZV group compared with the Ctrl group. dsDNA produced by VZV during replication, when recognized by the innate immune system, is known to activate core transcription factors, including nuclear factor kappa B, IRF3, and IRF7, which subsequently induces the expression of type I interferons (IFNα/β) and pro-inflammatory cytokines, to ultimately exert antiviral effects (Paludan et al., 2011). Innate immune cells also play an essential role in the whole immune process, and *in vitro* cultured monocytes and monocyte-derived macrophages produce pro-inflammatory cytokines such as IL-6, IL-8, and tumor necrosis factor alpha during VZV infection (Wang et al., 2005). The up-regulation of HLA molecules (e.g., HLA-A and HLA-DRB3) and cytotoxic T-cell markers (e.g., CD8A) indicates robust activation of viral antigen presentation and cytotoxic T-lymphocyte responses, which may contribute to both clearance of latent virus from infected neurons and immune-mediated tissue damage (Sato-Takeda et al., 2006; Colombo et al., 2021). Excessive or persistent activation of adaptive immunity may contribute to the development of neurological sequelae in some patients. Down-regulated pathways were significantly enriched for diminished intercellular adhesion and suppressed secretion and repair functions, indicating that these may be related to blood–brain barrier disruption, viral dissemination or immune escape, and dysregulation of anti-inflammatory and immune regulatory functions.

### 4.5 Cytokine signaling and the potential role of IL-9

Wikipathway enrichment analysis revealed that lymphocyte activation and inflammatory signaling pathways were significantly up-regulated in VZV CNS infection, with comprehensive up-regulation observed in T/B cell receptor signaling pathways and cytokine signaling pathways (e.g., IL-2/3/4/5/6/7/9). This aligns with previous reports of elevated inflammatory factors in the CSF of patients with VZV infection (Lind et al., 2019). Within this broad inflammatory milieu, IL-6, as a well-characterized pleiotropic cytokine, plays a central role in antiviral immunity; it not only enhances the early innate immune response by promoting neutrophil chemotaxis and autophagy (Wright et al., 2014; Croxford et al., 2014; Florentin et al., 2021), but also acts as a key regulator of adaptive immunity. IL-6 critically shapes the cellular immune response by synergizing with TGFβ to promote the differentiation of CD4+ T cells into Th17 cells while inhibiting their development into Treg cells, thereby finely tuning the Th17/Treg balance (Kimura and Kishimoto, 2010). Furthermore, it serves as a potent facilitator for B cell activation and antibody production. These combined actions establish IL-6 as a cornerstone of the host’s defense mechanism against viral infection. However, our data indicate that among the up-regulated cytokines, IL-9 exhibited the most pronounced change, followed by IL-7 and IL-4. This distinct pattern indicates that in the specific immune context of VZV CNS infection, beyond the generalized inflammation driven by IL-6, IL-9 may play a unique and critical role.

In our proteomic analysis, IL-9 was among the most prominently upregulated cytokines in the CSF of patients with VZV CNS infection. IL-9 is a pleiotropic cytokine produced by T helper 9 (Th9) cells that signals through the IL-9 receptor to activate JAK-STAT pathways (Chen et al., 2025). Although its role in viral CNS infection remains incompletely understood, studies in other neuroinflammatory conditions suggest that IL-9 may exert both pro-inflammatory and regulatory effects by modulating T cell responses, B cell activity, and glial cell function (Guadalupi et al., 2024; Elyaman et al., 2009).In the context of VZV CNS infection, the observed elevation of IL-9 may reflect an active neuroimmune response and could be associated with both antiviral defense and immunopathology. However, given the lack of direct evidence in VZV infection, these interpretations should be considered exploratory. Further studies are required to clarify the cellular sources, downstream targets, and functional significance of IL-9 signaling in this setting.

### 4.6 Complement activation in severe encephalitis: a case-based observation

One of our samples originated from a patient with severe encephalitis. The patient, a 73-year-old woman, presented with a clinical course of rapid progression from acute myelitis to severe encephalitis within three months. Her condition deteriorated sharply during the second admission, manifesting as high fever, impaired consciousness, mutism, and quadriparesis. Key laboratory findings indicated severe intracranial infection: cerebrospinal fluid (CSF) pressure was significantly elevated (210 mmH_2_O), protein levels were markedly increased (7.67 g/L), and an inflammatory cellular response predominantly consisting of lymphocytes was observed. mNGS of the CSF detected a high load of VZV sequences (119,800), providing high-confidence etiological evidence. Brain MRI revealed multiple new lesions and microhemorrhages in supratentorial, infratentorial, and brainstem regions, consistent with imaging features of severe viral encephalitis with vasculitic injury. Based on the critical clinical presentation, pronounced inflammatory markers, and definitive viral evidence, the case was ultimately diagnosed as severe VZV encephalitis and resulted in death. A comparison between groups revealed that the up-regulated proteins in the Severe encephalitis group were mainly focused on complement-associated proteins, including C3, C8A, C8G, C7, C6, and ITGAM, which are key components of the membrane attack complex, and an activated complement system increases endothelial and host cell damage (Kumar, 2020). The up-regulation of these complement-related proteins may reflect extensive cellular injury in the brain, and in severe VZV encephalitis, complement-mediated inflammatory injury may be associated with poor clinical outcomes. While complement-mediated damage has been implicated in endothelial and neuronal injury, the extent to which it contributes to disease severity in VZV encephalitis remains unclear. Given that this observation is based on a single case, it should be interpreted with caution.

### 4.7 Study limitations

This study has several limitations that warrant acknowledgment. The sample size for the proteomic analysis was relatively small, especially for clinical subgroups, and the results, although enlightening, need to be verified in a larger cohort. The study was observational, and the identified protein differences are correlative; their causality and specific functional roles need to be further verified.

## 5 Conclusion

This study integrates clinical and proteomic analyses to reveal the clinical and molecular features of VZV CNS infection. Synergistic over-activation of the type I interferon response, inflammatory signaling, and lymphocyte activation drives a robust neuroinflammatory reaction, which may underlie blood–brain barrier disruption and neurological deficits. Furthermore, over-activation of the complement system in severe encephalitis provides new insights for understanding disease severity and potential therapeutic targets. These findings underscore the potential of this approach for developing diagnostic markers and targeted therapeutic strategies.

## Data Availability

The authors declare that the data supporting the findings of this study are available within the paper and its supplementary information files. Should any raw data files be needed in another format they are available from the corresponding author upon reasonable request. Source data are provided with this paper.
